# The Influence of Consumption Purpose on Consumer Preferences for Fruit Attributes: The Moderating Effect of Color Perception

**DOI:** 10.3390/foods14111902

**Published:** 2025-05-27

**Authors:** Yihan Wang, Lingying Liu, Yangyang Wei

**Affiliations:** 1 School of Art, Wuhan Business University, Wuhan 430056, China; 2 Architecture and Design College, Nanchang University, Nanchang 330031, China

**Keywords:** fruit, color perception, consumer preferences, consumption purpose, health awareness

## Abstract

With the increasing awareness of health among residents, consumers are paying more attention to their eating purposes and food safety when choosing fruits. This study aims to explore the impact of eating purpose on consumers’ preferences for fruits and fruit products under the mediation of color perception. The study obtained experimental data from 489 urban consumers in China through the Credamo data collection platform. Furthermore, four experimental groups were set up to propose six hypotheses based on the influence of eating purpose on consumer preferences for fruits and their products. The study utilized Likert scale questionnaires, chi-square tests, and variance analysis for data mining and cross-validation. The results indicate that the visual characteristics of fruits (especially color) affect the purchase preferences of consumers with different eating purposes. Approximately 65% of health-oriented consumers are highly sensitive to the color and nutritional value of fruits. They believe that fresh fruits are rich in natural nutrients and play an important role in maintaining health and preventing diseases. Meanwhile, around 62% of consumers with specific nutritional needs prefer processed fruit products, such as fruit preserves or dried fruits. These consumers have a weaker perception of color and focus primarily on the functionality of the fruits. Additionally, the study found that safety/taste preferences acted as a mediator and associative learning as a moderating variable. Around 58% of consumers indicated that their purchase preferences are influenced by safety and taste, and the relative importance of safety and taste preferences significantly mediated the relationship between eating purpose and purchase preferences. Under the moderating effect of associative learning, health-oriented consumers, when associative learning is activated, are about 45% more likely to choose fresh fruits. The study highlights consumers’ health-conscious perceptions in fruit selection, focusing on how color perception moderates the preference choices of different consumer groups based on their eating purposes. It emphasizes the need for businesses to adjust product positioning and marketing strategies according to consumer perceptions to promote broader healthy eating behaviors.

## 1. Introduction

The World Health Organization (WHO) recommends that consuming an adequate level of fruits is one of the essential elements of a healthy diet, with fresh fruits being a vital source of essential vitamins, minerals, and antioxidants for the human body. Along with the popularization of healthy eating concepts, consumers are increasingly likely to prefer foods such as fruits and vegetables, as they are aware of their potential preventive effects on certain non-communicable diseases [1]. Fruits, as foods rich in a variety of nutrients, with excellent sensory characteristics and extensive health benefits, are an important part of achieving a scientific diet structure, ensuring balanced nutrient intake, and maintaining healthy eating habits [2]. Although fruit consumption is crucial for a balanced diet and fruit consumption has been increasing accordingly, the global fruit consumption level remains below the recommended amount (200–300 g per day) [3,4]. A systematic analysis conducted by Micha et al. across 266 countries and regions indicated that the global average fruit consumption in 2010 was 81.3 g per day [5], and by 2019, the global average fruit consumption was approximately 150–200 g per day, but there were significant regional differences. Moreover, the Global Burden of Disease Study in 2017 revealed that insufficient fruit intake was one of the top three dietary risk factors leading to death and disability, with 2.42 million deaths attributed to insufficient fruit intake [6], highlighting the urgent need for global coordinated efforts to improve healthy eating practices.

Several studies, both domestic and international, have shown that increasing fruit intake can reduce the incidence and mortality rates of cardiovascular diseases, digestive system cancers, and other conditions [7,8]. National dietary guidelines recommend that each person should consume 400–800 g of fruits and vegetables daily. Based on global fruit and vegetable production, average per capita consumption increased from 306 g per day in 2000 to 390 g per day in 2017 [9,10,11], still falling short of the target value. Since fruits can be categorized into processed (e.g., fruit preserves, canned fruits, dried fruits, fruit jams, etc.) and unprocessed forms, the nutrients they contain differ. In addition to purchasing fresh fruits, Zhao Xin et al. [12] conducted a survey on consumer purchases of processed fruit products and found that more than half of consumers buy processed fruit products, while only 3.93% of consumers do not. With the addition of food additives (such as colorants, flavorings, preservatives, etc.) to food production, consumers are provided with the “convenience” of consumption and storage [13]. Although more and more consumers seem to prefer fresh, natural, and unprocessed fruits [14,15], it is interesting that under certain circumstances, consumers are more willing to consume processed fruits. Zhang Tianqi analyzed health data from 100,000 people collected between 1986 and 2009 and found that the consumption of freshly squeezed juice was positively correlated with an increased risk of diabetes, while the consumption of specific low-sugar fruits showed the opposite effect [16]. Wang Linlin et al. found that dried fruits have positive effects on blood sugar control, cardiovascular health, and bone health, making them a healthy snack option [17]. Research by Keast D R et al. showed that only 6.9% of all consumers had a habit of eating dried fruits [18]. Guo Qiya et al. analyzed and concluded that the intake of fresh fruits among people aged 60 and above in China is severely insufficient, and efforts should be made to increase fresh fruit consumption to improve health and actively promote healthy aging [19]. Martínez-Carrasco et al. surveyed a representative sample of Spanish consumers and found growing interest in choosing healthier and more natural foods, with a preference for fruits without preservatives [20]. Existing research indicates that various factors influence fruit consumption, with consumer behavior often associated with consumer perceptions, gender differences, cost, taste preferences, color, purchase intentions, and decision-making processes [21,22,23,24]. Wahyuningtias, D. et al. used SPSS software (Statistics 27.0.1) to analyze survey results and explore consumers’ acceptance and preferences for innovative strawberry jam slices [25]. Wei Yongyi et al. analyzed the sensory characteristics of apple sauce using t-tests and quantitative descriptive analysis, providing an effective method for evaluating apple sauce quality [26]. Han Donghai et al. used variance analysis to investigate the relationship between black-heart disease in Ya pears and differences in the fruit surface color, sugar content, and firmness [27].

By reviewing research on how color perception influences consumer preferences for fruits, it is found that consumers’ health-conscious perceptions in fruit selection are not sufficiently explored, and businesses struggle to adjust food positioning and marketing strategies based on consumer perceptions. This study focuses on how color perception moderates the effect of consumption purpose on consumers’ preferences for fruits and their products, using variance analysis to explore the degree and significance of its impact on consumer preferences. The study provides a new perspective and approach for research in fields such as fruit and vegetable consumption and processing, offering both theoretical and practical support for consumer behavior choices, with the aim of promoting broader healthy eating behaviors.

## 2. Materials and Methods

### 2.1. Experimental Subjects

To test the research hypotheses, the study recruited participants between July and September 2024 through the Credamo data collection platform, conducting an online survey with 500 urban consumers in China to collect survey data. Participation in the experiment was voluntary and anonymous. Participants were informed that the survey aimed to explore consumer preferences for fruit purchases and did not involve any personal sensitive privacy data or generate any commercial interests. After excluding respondents who provided inconsistent data or failed to complete the survey, 489 consumers participated in the experimental survey. The sample covered consumers from different regions, and quota sampling was used based on gender, age, and education level [28]. The sociodemographic data of the sample population, measurements of physical health, and food preferences, as shown in Table 1, were collected. The advantages of this survey method include low cost, quick feedback, wide coverage, and no limitations regarding time or space [29].

Experiment 1 randomly recruited 200 consumers to participate in an experiment on “The Impact of Consumer Eating Purpose on Preferences for Fruits and Fruit Products”, which included 44.5% males, 54% females, and 1.5% unspecified. Experiment 2 recruited 400 consumers to participate in the experiment, including 35.5% males, 63.5% females, and 1% unspecified, to compare purchase intentions under different eating purposes. To verify whether safety/taste preferences serve as a mediating variable in consumers’ purchase decisions, Experiment 3 randomly recruited 200 consumers, including 39% males, 60% females, and 1% unspecified. Experiment 4 recruited 400 consumers to investigate the importance of the moderating effect of associative learning on consumer decision-making, which included 49.5% males, 46.6% females, and 3.9% unspecified.

### 2.2. Material Preparation Techniques and Methods

The study uses conjoint analysis to evaluate various attributes (features, functions, or characteristics) and their impact on consumer preference. Conjoint analysis is a multivariate statistical analysis method used to study consumer product preference choices [30]. This method is applied to evaluate fruits and their products, classifying fruits into processed and unprocessed categories based on their functions and characteristics. Processed fruits are those that undergo a series of physical, chemical, or biological treatments to enhance flavor, improve nutritional value and economic benefits, and effectively extend the availability of fruits. These can be mainly categorized into five types: preserved fruits, canned fruits, fruit juices, dried fruits, and fruit jams [31]. To ensure the orthogonality of the Conjoint Analysis, we defined unique attributes and their levels for each category and ensured that these attributes were logically independent of one another, with no cross-influence. For example, preserved fruits may include attributes such as sweetness, texture, and types of additives; canned fruit attributes may include packaging form and sugar content; fruit juice attributes may include juice concentration, sugar content, and pulp presence; dried fruit attributes may include processing method and texture; and fruit jam attributes may include jam concentration, etc., to accurately assess the independent contribution of each attribute to consumer preferences.

**Figure 1:**
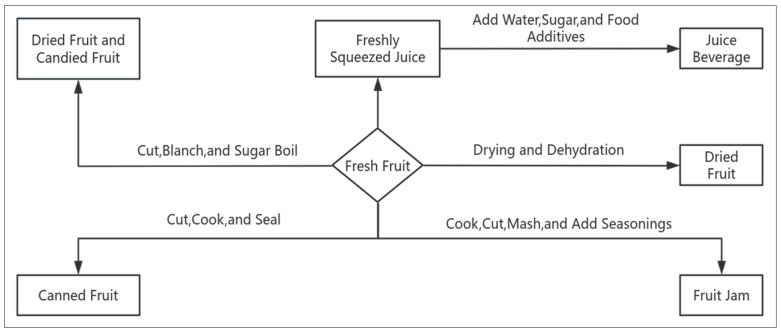
Common Fruit Processing Techniques and Classifications.

The preservation process for preserved fruits retains some of the fruit’s original flavor but adds large amounts of sugar, using high-concentration sugar syrup to lower water activity, which prevents microbial activity due to physiological dehydration [32]. The canning process preserves the original flavor and nutrients of fruits but typically adds sugar and preservatives. Canned fruit products have been popular in the international market for nearly a century, due to their convenience for carrying and eating, long shelf life, and ability to regulate market demand across different seasons, making them highly favored by consumers [33]. The juice extraction process involves pressing and filtering the fruit juice, followed by the addition of water, sugar, and food additives to adjust the flavor, creating fruit juice products. The drying process uses methods such as air drying, sun drying, baking, and vacuum drying to “extract” the moisture from fresh fruits, turning them into dried products [34]. This process retains most of the fruit’s nutritional content and original flavor, typically without the addition of preservatives. Fruit jams are made by cooking or chopping the fruit, then mashing it and adding flavoring agents to form a jam-like consistency, which is then sealed for preservation. The techniques and classifications are shown in Figure 1.

### 2.3. Experimental Design

Considering the characteristics and nutrients of fruits, the study categorizes them based on key nutrients such as Vitamin A, Vitamin B, Vitamin C, Vitamin E, carotenoids, and lutein [35], with varying nutritional compositions and effects. To ensure the validity of the experiment, the study selected fruits rich in different nutrients for the verification of the four experiments, namely oranges, kiwis, grapes, and fresh blueberries, as shown in Table 2.

To study consumer preferences for fruits and their products, the research set up experiments on the purchasing behavior of fresh fruits versus processed fruits. In the experiment, respondents were required to make preference choices within four different experimental groups and situational settings. Given that respondents’ decisions may be influenced by various factors, such as the nutrients contained in the fruits, fruit attributes, and fruit colors, the study selected typical fruits and common fruit processed products for experimentation to test the hypotheses (Table 3).

The experimental sample was selected from the following four types of fresh fruits and processed fruit products:

Experiment 1: Fresh orange-colored oranges rich in Vitamin C and light yellow canned oranges.

Experiment 2: Green kiwis rich in Vitamin C and yellow-green kiwi preserves.

Experiment 3: Green grapes rich in Vitamin B and yellow-green dried grapes.

Experiment 4: Deep blue blueberries rich in Vitamin E and purple-black blueberry jam.

### 2.4. Experimental Hypotheses

Therefore, the study divides the fruit consumption population into two main groups: one is the health-oriented eating purpose, where consumers focus on the naturalness and health benefits of fruits and are highly sensitive to the color, freshness, and nutritional value of fruits. The other group is the specific nutritional needs-oriented eating purpose, where consumers require specific nutrients due to particular health needs (such as weight loss, muscle gain, blood sugar control, etc.) and tend to be less sensitive to color, focusing more on nutritional content and functionality.

In the experiment, two types of fruits will be shown to the respondents: one is a fresh fruit rich in the nutrients they need, with softer and more coordinated colors. This natural color gives a visual sense of freshness and vitality, instinctively associating it with health. The other is a processed fruit that may contain the nutrients they need but has colors altered by artificial dyes, possibly lacking the soft and harmonious hues of fresh fruit, or appearing too bright due to the dyes, creating some visual strain for the respondents. Finally, the respondents will choose their preferred fruit based on these options.

Based on the above experimental setup, the study proposes the following hypotheses:

**H1**:  *Consumers with health-oriented (specific nutritional needs-oriented) eating purposes prefer unprocessed (processed) fruits.*

**H1a**:  *When the consumer’s eating purpose is health-oriented, compared to processed fruits, they are more likely to prefer unprocessed fruits with softer, coordinated colors.*

**H1b**:  *When the consumer’s eating purpose is specific nutritional needs-oriented, compared to unprocessed fruits, they are more likely to prefer processed fruits with low-saturation colors or vibrant hues.*

**H2**:  *The relative importance of safety and taste preferences mediates the relationship between consumers’ eating purposes and their preferences for fruit attributes.*

**H3**:  *Consumers’ associative learning moderates the effect of eating purpose on their fruit attribute preferences.*

**H3a**:  *When associative learning is activated, consumers’ existing perception that “processed fruits = nutrient loss” is triggered (consuming processed fruits is unhealthy), and the health-conscious consumption purpose positively influences consumers’ preference for fresh fruits.*

**H3b**:  *When associative learning is not activated, the effect of eating purpose on the consumer’s preferences does not exist.*

### 2.5. Experimental Process

First, to ensure the validity of the experimental results, the study implemented some manipulations with the respondents. This involved controlling the variables related to consumers’ consumption purposes and the types of fruits and their products, setting different scenarios for the participants to obtain experimental data feedback under varying conditions. The respondents’ reactions were then examined to ensure that the independent variables were successfully manipulated. To verify whether there is a correlation between the two variables, the study used the chi-square test. This test compares two or more frequencies to detect the difference between the actual frequencies at a certain significance level and the expected frequencies based on a theoretical model or distribution characteristics [39]. In this case, $O_{i}$ represents the actual frequency, and $E_{i}$ represents the expected frequency. The calculation process was completed using SPSS software (version 28.0), with the following formula:

(1) $$X^{2}=\sum \frac{{\left(O_{i}-E_{i}\right)}^{2}}{E_{i}}$$

Secondly, the study used a seven-point Likert scale to have the respondents indicate their preferences for fruit attributes. The Likert scale is a commonly used survey design method that includes options such as “strongly prefer”, “prefer”, “slightly prefer”, and “neutral”, converting qualitative attitudes into quantitative data [40]. The feedback data from the respondents were statistically analyzed to determine the distribution of their satisfaction levels.

**Figure 2:**
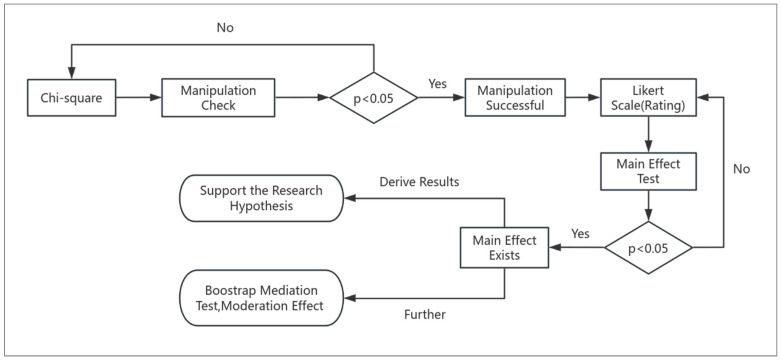
Experimental Process Design.

Finally, the study conducted main effect testing on the survey results, which included one-way analysis of variance (ANOVA), two-way ANOVA, and covariance analysis. Main effect testing is used to determine whether there are significant differences between three or more population means and utilizes within-group and between-group differences to construct statistical measures [41]. After confirming the existence of main effects, the study further used the PROCESS plugin in SPSS to perform Bootstrap mediation testing (to determine if X affects Y through the mediator M). The PROCESS software (Statistics 27.0.1) is a tool that helps us perform such decompositions. It first calculates the direct effect of X on Y, then checks if X activates M, which subsequently leads to Y. Finally, it uses effect size multiplication and confidence intervals to determine whether M truly serves as a bridge that mediates the mutual influence between X and Y. And moderation effect testing (to assess whether a third variable M influences the relationship between the independent variable X and the dependent variable Y) [42]. The overall experimental process is shown in Figure 2.

## 3. Results

The study designed four experiments to verify consumers’ preference choices for fruits and their products, highlighting the moderating effect of color perception. Experiment 1 used one-way analysis of variance (ANOVA) to verify the impact of consumers’ eating purposes on their preferences for fruits and their products. Experiment 2 used two-way ANOVA to verify consumers’ purchasing intentions under different eating purposes, examining their preferences for fruits and their products. Experiment 3 employed one-way ANOVA and mediation testing to verify the mediating role of the relative importance of safety and taste preferences. Experiment 4 introduced associative learning [43], and variance analysis was used to test and verify the moderating effect of this factor.

### 3.1. Experiment 1: The Influence of Consumption Purpose on Preferences for Fruits and Fruit Products

Experiment 1 used a one-way between-subjects design to test Hypothesis H1. The experiment selected orange-colored oranges rich in Vitamin C and light yellow canned oranges. All participants were randomly assigned to two groups, each participating in either the health-oriented eating purpose or the specific nutritional needs-oriented eating purpose test, in order to compare differences between the groups.

The study first ensured the validity of the experiment’s results by conducting a manipulation check of consumers’ eating purposes. In the health-oriented purpose scenario group, participants were asked to imagine: “Currently, your physical condition is good, and all your nutritional indicators are balanced, but you want to further enhance your health by eating fruits rich in Vitamin C”. In the specific nutritional needs-oriented purpose scenario group, participants were asked to imagine: “Currently, your physical condition has certain deficiencies, and you want to alleviate your health condition by eating fruits rich in Vitamin C”. Data analysis was performed using SPSS software, with variables inputted and a contingency table designed (see Table 4 and Table 5) [44]. Finally, the chi-square test results, shown in Table 6, indicated that the vast majority of participants (90.5%) successfully passed the manipulation check for consumer eating purposes. Significant differences were observed between the different eating purposes (*X*^2^ = 7.037, p=0.014<0.05), confirming the success of the manipulation of eating purposes.

**Figure 3:**
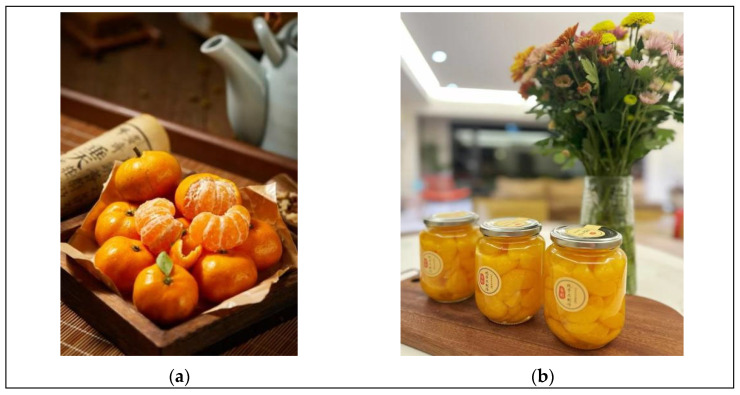
(**a**) is Orange-yellow Fresh Oranges and (**b**) is Light Yellow Canned Oranges.

All participants were asked to measure their preferences for fruit attributes (fresh fruit vs. processed fruit) using a seven-point Likert scale. The experiment selected two types of fruits rich in Vitamin C: one was a vibrant orange-colored fresh orange, as shown in Figure 3a, and the other was a light yellow canned orange with lower saturation, as shown in Figure 3b. The hypothesis was: “These two Vitamin C-rich fruits are priced the same, and you plan to have one as a post-meal snack”. Participants were asked to rate their preferences for the two fruits and were provided with the following scale: 1 = strongly prefer the fresh orange, 2 = moderately prefer the fresh orange, 3 = slightly prefer the fresh orange, 4 = neutral, 5 = slightly prefer the canned orange, 6 = moderately prefer the canned orange, and 7 = strongly prefer the canned orange.

A one-way analysis of variance (ANOVA) was conducted with consumers’ eating purposes as the independent variable and preferences for fruits and their products as the dependent variable. The results showed that consumers’ eating purposes had a significant effect on their purchasing intentions. Compared to the health-oriented eating purpose, participants with a specific nutritional needs-oriented eating purpose preferred canned oranges, as shown in Table 7.

In this context, the analysis showed that (*M_Health-orinted_* = 2.55, ${SD}_{Health\text{-}oriented}=1.234$; *M_Specific-Nutritional-Needs_* = 3.85, ${SD}_{Specific\text{-}Nutritional\text{-}Needs}=1.565$; F=8.506, *p* = 0.006 < 0.05), indicating that the main effect of eating purpose exists, creating a difference in consumer preference choices, which confirms Hypothesis H1. It should be noted that Experiment 1only measured the impact of consumer preferences for fruits and their products, without specifically comparing the differences in purchase intentions underdifferent consumption purposes. Therefore, in Experiment 2, the study willconsider both types of consumption purposes, while still focusing on processedand unprocessed fruits rich in Vitamin C, and will replace the researchsubjects with fresh fruits and preserved fruits for the experiment.

### 3.2. Experiment 2: Consumer Preference for Purchase Intentions of Fruits and Their Products

**Figure 4:**
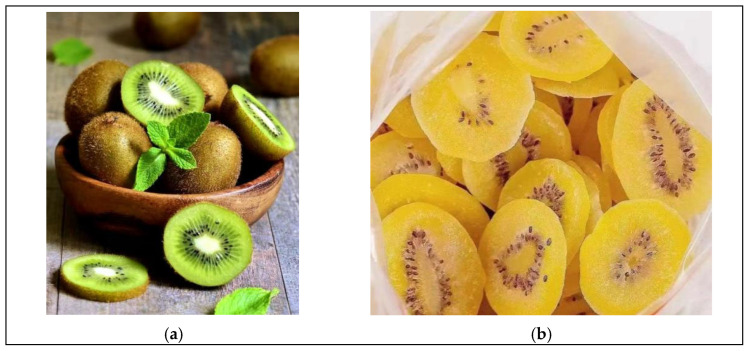
(**a**) is Green Kiwi and (**b**) is Yellow-Green Kiwi Fruit Preserves.

In Experiment 2, a two-factor between-subjects design was used to test Hypotheses H1a and H1b, and to further validate Hypothesis H1 based on the findings from Experiment 1. The experiment used Vitamin C-rich green kiwis and yellow-green kiwi preserves, as shown in Figure 4. It employed a 2 (health-conscious vs. specific nutritional needs) × 2 (kiwi vs. kiwi preserve) between-subjects design, with participants randomly assigned to one of four groups.

The experiment manipulated the consumption purpose and preferences for fruits and fruit products to ensure the validity of the results. In the brightly colored yellow-green kiwi preserve (processed fruit) group, participants were shown the following content: “To meet your nutritional needs, assume you are an athlete or fitness enthusiast who requires high-sugar foods. Compared to fresh kiwi, kiwi preserves provide higher energy and sugar”. In the fully colored green kiwi (fresh fruit) group, participants saw: “To meet your nutritional needs, assume you are pursuing a natural and healthy diet and want to intake rich vitamins and minerals daily. Compared to kiwi preserves, fresh kiwi is rich in natural Vitamin C and dietary fiber”. Participants were then asked to rate their preferences, with the following scale: 1 = strongly prefer fresh kiwi, 2 = somewhat prefer fresh kiwi, 3 = slightly prefer fresh kiwi, 4 = neutral, 5 = slightly prefer kiwi preserve, 6 = somewhat prefer kiwi preserve, 7 = strongly prefer kiwi preserve.

The manipulation check results are shown in Table 8. The vast majority of participants (93.8%) passed the manipulation check for consumption purpose, with significant differences across different consumption purposes (*X*^2^ = 7.521, p=0.007<0.05), indicating that the manipulation of consumption purpose was successful. As for the manipulation check of fruit attribute preferences, as shown in Table 9, the results indicate (F=10.654, *p* = 0.002 < 0.05), meaning that the manipulation of fruit and product preferences was successful.

The measurement of participants’ purchase intention was conducted with the question: “How likely are you to purchase kiwi fruit preserves?” (1 = very unlikely, 5 = very likely, with higher values indicating a greater likelihood of purchase). A two-way ANOVA was performed with consumer eating purpose and fruit attribute preference as the independent variables, and purchase intention as the dependent variable, as shown in Table 10 [45].

The output from the analysis reveals a significant interaction between eating purpose and fruit and product preference (*F* = 4.968, p=0.029<0.05). Further simple effects analysis, through pairwise comparisons, yields the following results:

Consumers with a health-oriented purpose are more inclined to purchase fresh fruit. Specifically:

*M_Kiwi_* = 3.50, ${SD}_{Kiwi}=0.304$; $M_{Kiwi\text{-}Preserves}=1.632$, *SD_Kiwi-Preserves_* = 0.156; F=29.867, p<0.001.

Consumers with a specific nutritional needs-oriented purpose are more inclined to purchase processed fruit. Specifically:

$M_{Kiwi}=2.857$, ${SD}_{Kiwi}=0.364$; $M_{Kiwi\text{-}Preserves}=3.548$, ${SD}_{Kiwi\text{-}Preserves}=0.173$; *F* = 2.949, p<0.001.

**Figure 5:**
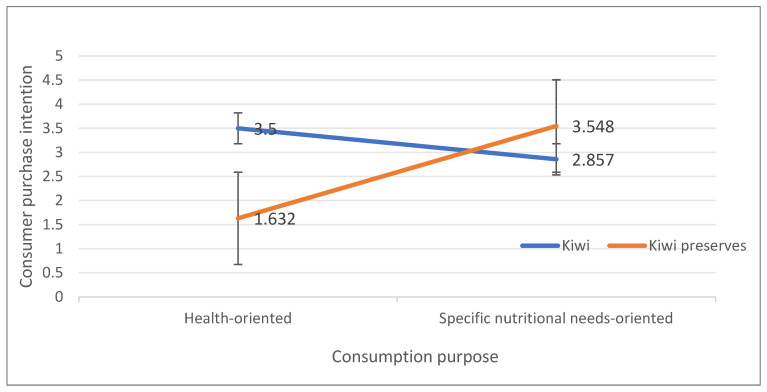
The Effect of Eating Purpose on Consumer Purchase Intentions.

These experimental results further validate hypotheses H1a and H1b and provide support for hypothesis H1, as shown in Figure 5.

### 3.3. Experiment 3: The Mediating Role of Safety and Taste Preferences in Consumer Purchase Decisions

**Figure 6:**
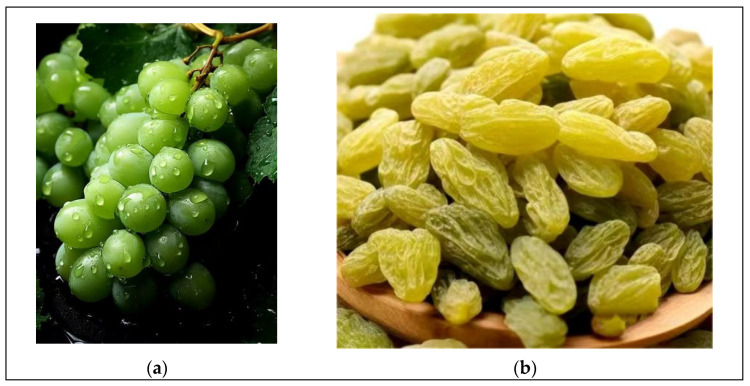
(**a**) is Green Grapes and (**b**) is Yellow-Green Raisins.

To further validate the mediating role of safety and taste preferences in consumer purchasing, this experiment selected Vitamin B-rich green grapes and yellow-green raisins, as shown in Figure 6. The experiment employed a one-factor between-subjects design, with all participants randomly assigned to either the health-conscious or specific nutritional needs consumption purpose groups. Experiment 3 referenced the consumption purpose manipulation check from Experiment 1. The chi-square test results, as shown in Table 11, indicate that the vast majority of participants (91%) passed the manipulation check for consumption purpose, with significant differences across different consumption purposes (*X*^2^ = 5.025, p=0.025<0.05), meaning that the manipulation of consumption purpose was successful.

Using consumption purpose as the independent variable and fruit attribute preference as the dependent variable, a one-way ANOVA was conducted on the sample. The results are shown in Table 12. Consumption purpose has a significant effect on purchase intention. Compared to health-conscious consumers, those with specific nutritional needs were more inclined to prefer raisins. Therefore, it can be concluded that:

*M_Health-orinted_* = 2.93, ${SD}_{Health\text{-}oriented}=1.507$; *M_Specific-Nutritional-Needs_* = 4.50, ${SD}_{Specific\text{-}Nutritional\text{-}Needs}=1.469$; F=13.229, p≤0.001; the results further support Hypothesis H1.

**Figure 7:**
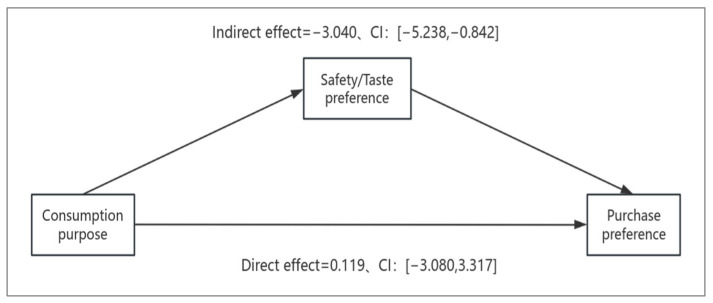
Mediating Effect of Safety/Taste Preference.

The relative importance of safety/taste preferences was measured by participants (when considering purchasing fruit, do you prioritize the safety of fresh grapes or the unique taste of raisins? 1 = taste preference, 7 = safety, with higher values indicating a stronger focus on fruit safety benefits). The mediating effect was tested using the PROCESS plugin in SPSS [46], employing the Bootstrap method for mediation effect testing (Model 4, sample size 5000, CI 95% confidence interval) [47]. With consumption purpose as the independent variable, consumer purchase preference as the dependent variable, and safety/taste preferences’ relative importance as the mediator, as shown in Figure 7, the mediation model was used to clarify the dynamic interaction between these variables [48]. The indirect effect was −3.040, SE=1.121, 95% CI: [−5.238, −0.842], which does not contain 0, indicating that safety/taste preferences significantly mediated the relationship between consumption purpose and purchase preference. When controlling for the mediating variable, the effect of consumption purpose on purchase preference was not significant, with a direct effect of 0.119, SE=1.632, 95% CI: [−3.080, 3.317], which includes 0, as shown in Table 13. This validates Hypothesis H2.

### 3.4. Experiment 4: The Moderating Effect of Associative Learning

**Figure 8:**
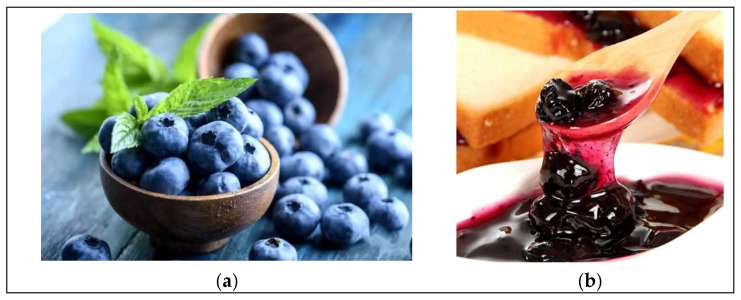
(a) is Deep blue blueberries and (b) is purple-black blueberry jam.

Experiment 4 introduces associative learning to test and validate the moderating effect of associative learning on participants. Vitamin E-rich fruits were selected for the experiment, using a 2 (processed fruit vs. unprocessed fresh fruit) × 2 (associative learning: activated vs. non-activated) between-subjects design. Participants were randomly assigned to one of the four groups. The experimental materials included soft-colored deep blue blueberries and brightly colored, eye-catching purple-black blueberry jam (Figure 8).

**Figure 9:**
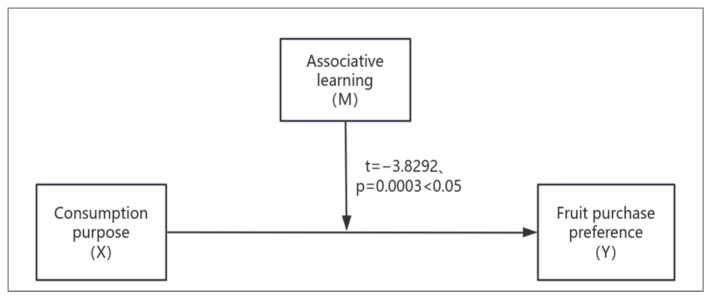
Moderating Effect Model of Associative Learning.

Based on health cognition research, participants may engage in associative learning, such as associating certain processed fruits with additives and nutrient loss, which leads to unhealthy perceptions and affects consumer purchase intentions. Therefore, the participants were divided into two groups: the associative learning activation group and the non-activation group. In the associative learning activation group, participants were shown brightly colored blueberry jam and informed about the common perception that processed fruits may contain additives that cause significant nutrient loss. In the non-activation group, participants were only shown the blueberry jam without any indication of the relationship between nutrient loss and additives. With consumer consumption purpose as the independent variable (X), purchase preference (7-point Likert scale: 1 = strongly prefer blueberries, 7 = strongly prefer blueberry jam) as the dependent variable (Y), and associative learning (activated = 1, non-activated = 2) as the moderating variable (M), a moderation effect model (Model 1) was constructed, as shown in Figure 9.

Using a two-way ANOVA, the analysis results show:

In the associative learning activation group,

$M_{activation/Health\text{-}oriented}=1.810$, $M_{activation/Specific\text{-}Nutritional\text{-}Needs}=3.444$;

In the associative learning non-activation group,

*M_non-activation/Health-oriented_* =4.069, *M_non-activation/Specific-Nutritional-Needs_* = 4.545

Furthermore, the *p*-values for consumption purpose (X), associative learning (M), and the interaction term (X * M) were all less than 0.05 (Table 14). It is evident that when associative learning was activated, the effect of consumption purpose on fruit attribute preferences became significant. Consumers, when purchasing blueberries or blueberry jam, associated the possibility of additives in the blueberry jam leading to nutrient loss, creating an unhealthy perception. As a result, they were more inclined to purchase fresh blueberries, thus confirming Hypothesis H3.

## 4. Discussion

### 4.1. Descriptive Statistical Results Analysis

Existing research indicates that overweight, obese individuals, or those with diabetes tend to prefer low-sugar or sugar-free dried fruits [17]. Furchner-Evanson et al. found that in a dietary intervention for overweight women, the glycemic response and insulin curve were significantly reduced after consuming dried plums [49]. Dong Yang’s research showed that the glycemic index (GI) values of dried apples, red dates, raisins, and dried figs were significantly lower than white rice, helping to reduce the maximum fluctuation of blood glucose levels [50]. Athletes or fitness enthusiasts are more inclined to choose high-energy, high-protein canned fruits or fruit preserves. Based on this, the study further constructs a research framework using various theoretical methods such as chi-square tests and variance analysis. The chi-square test was effectively applied to compare the preference distribution differences between different consumer categories, while variance analysis was used to explore the degree and significance of various factors influencing consumer preferences, providing new perspectives and approaches for research in related fields. The study found that when consumers have a health-conscious eating purpose, they are more sensitive to the color and nutritional value of fruits and are more likely to purchase unprocessed fresh fruits. On the other hand, when consumers have specific nutritional needs as their eating purpose, they are less sensitive to color and focus more on functionality, making them more inclined to purchase processed fruits. It is noteworthy that the relative importance of safety and taste preferences mediates the relationship between consumers’ eating purposes and their preferences for fruits and their products [51].

The study validated the positive impact of fresh fruits on health-conscious consumers through four experiments, examining the mediating role of safety/taste preferences and the moderating effect of associative learning. In Experiment 1, the preferences of consumers for Vitamin C-rich oranges and orange preserves were measured. Health-conscious (specific nutritional need) consumers preferred fresh oranges (oranges in preserves). Experiment 2 measured consumer preferences for Vitamin C-rich kiwis and kiwi preserves under different consumption purposes, revealing that health-conscious consumers tended to prefer fresh kiwis, while those with specific nutritional needs were more inclined to purchase kiwi preserves. Experiment 3 tested consumer willingness to buy Vitamin B-rich grapes and raisins, finding that safety/taste preferences significantly mediated the relationship between consumption purpose and purchase preference. When controlling for the mediating variable, the effect of consumption purpose on purchase preference became insignificant. Due to life experience, consumers may associate the consumption of fruit additives with a perception that “processed fruits = nutrient deficiency”, which leads to the intuitive belief that “fresh fruits are green and healthy”. Experiment 4 introduced associative learning, showing that when activated, associative learning significantly affected health-conscious consumers’ preference for fresh blueberries over blueberry jam.

#### 4.1.1. Discussion of Consumers’ Consumption Purpose on Fruit and Product Preferences

In Experiment 1, a manipulation of consumers’ consumption purpose was conducted, with 100 participants selected from each of the two groups. Participants were asked to imagine different scenarios, and the chi-square test yielded a result of *p* = 0.014 < 0.05, indicating that the manipulation of consumption purpose was successful. A one-way ANOVA was used to explore the effect of consumption purpose on preferences for fruits and fruit products. The experiment used Vitamin C-rich oranges and orange preserves as the experimental materials. A seven-point Likert scale was employed to collect participants’ preference data for fresh oranges and orange preserves. Health-conscious consumers generally believe that fresh fruits are rich in natural nutrients, meeting daily dietary needs for vitamins, minerals, and dietary fiber. As a result, they tend to prefer fresh fruits. In contrast, consumers with specific nutritional needs may require certain types or forms of fruit products due to health conditions. The results showed F=8.506, p=0.006<0.05, indicating the main effect of consumption purpose. Consumers with a health-conscious consumption purpose were more inclined to purchase fresh oranges, while those with a specific nutritional needs consumption purpose preferred orange preserves. This finding not only validates Hypothesis H1, which suggests that health-conscious (specific nutritional needs) consumers prefer unprocessed (processed) fruits, but it also further reveals the intrinsic link between consumption purpose and preferences for fruits and their products when making purchasing decisions.

#### 4.1.2. Discussion of Consumer Willingness to Purchase Fruits and Products

Building on Experiment 1, Experiment 2 further validated the hypothesis by selecting 117 health-conscious consumers and 93 consumers with specific nutritional needs. A manipulation experiment for consumption purpose and preferences for fruits and their products was conducted, yielding *p*-values less than 0.05, ensuring the validity of the experimental results. Subsequently, a two-way ANOVA was conducted to examine the willingness to purchase Vitamin C-rich kiwis and kiwi preserves among the two different consumption purpose groups. When measuring participants’ purchase intentions using the Likert scale, it was found that participants who did not identify their consumption purpose exhibited some randomness in their choice of fruits and fruit products, highlighting the importance of consumption purpose in consumer fruit selection behavior. The data results showed F=4.968, p=0.029<0.05, indicating a significant interaction effect between consumption purpose and preferences for fruits and fruit products. Consumers with different consumption purposes exhibited clear preference differences in their fruit and fruit product choices. This experimental result further validated Hypothesis H1a, which suggests that when the consumer’s consumption purpose is health-conscious, they are more likely to choose unprocessed fruits with soft, coordinated colors compared to processed fruits. It also validated Hypothesis H1b, which indicates that when the consumer’s consumption purpose is for specific nutritional needs, they are more inclined to purchase processed fruits with low saturation or vivid hues compared to unprocessed fruits. Additionally, this experiment provides positive support for the validation of Hypothesis H1 from Experiment 1.

#### 4.1.3. Discussion of the Mediating Role of Safety and Taste Preferences

Compared to Experiment 1 and Experiment 2, Experiment 3 maintained experimental design consistency while enhancing rigor and richness by replacing the nutrients in the fruits and their products, in order to further validate the generalizability of the research hypothesis. The study selected Vitamin B-rich green grapes and yellow-green raisins as the experimental materials, and an online survey was conducted with 200 urban Chinese consumers. A one-way ANOVA was conducted with consumption purpose as the independent variable and fruit and product preferences as the dependent variable. The results showed F=13.229, p<0.001, supporting Hypothesis H1. In Experiment 3, it was noted that there was a close interaction between consumption purpose, fruit and product preferences, and safety/taste preferences. On one hand, consumption purpose directly influenced participants’ preferences for fruits and their products; on the other hand, safety and taste preferences further moderated the relationship between consumption purpose and fruit/product preferences. This complex interaction mechanism reveals the diversity and complexity of consumer food choices. Therefore, safety and taste preferences were introduced as mediating variables, with consumption purpose as the independent variable and consumer purchase preferences as the dependent variable. Mediation effect testing was conducted to explore their role in the consumer decision-making process. The experimental results validated Hypothesis H2, which states that the relative importance of safety and taste preferences mediates the relationship between consumption purpose and preferences for fruits and their products. The data showed a direct effect of 0.119. After controlling for the mediating variable, the direct effect of consumption purpose on purchase preference became insignificant, further confirming the important role of safety and taste preferences in the consumer decision-making process.

#### 4.1.4. Discussion of the Moderating Effect of Associative Learning

In Experiment 4, associative learning was introduced as a moderating variable, and the study used vitamin E-rich blueberries and blueberry jam as experimental materials. In the associative learning activation group, the effect of consumption purpose on fruit preference was significant (p<0.05). Considering the negative association of additives in blueberry jam leading to nutrient loss, participants in this group tended to prefer fresh blueberries. However, in the non-activation group, where no associative learning cues were provided, the effect of consumption purpose on purchase willingness was lower. These results underscore the important role of associative learning in consumer decision-making, supporting Hypothesis H3, which states that associative learning moderates the effect of consumption purpose on consumers’ fruit attribute preferences.

When associative learning is activated, consumers associate “processed fruits = nutrient loss”, which leads health-conscious consumers to prefer fresh fruits. In the experiment, consumers with health-conscious purposes, upon seeing blueberry jam, received cues about “processed fruits potentially containing additives and nutrient loss”, prompting a significant preference for fresh blueberries. This finding supports Hypothesis H3a, where the activation of associative learning makes health-conscious consumers more likely to select fresh fruits over processed ones. When associative learning is not activated, consumption purpose does not significantly influence consumer preferences for fruits, as observed in the control group. Without associative learning cues, consumers did not show significant preference differences between fresh fruit and processed fruit products, validating Hypothesis H3b.

Experiment 4, closely linked to the previous experiments, further deepened our understanding of consumer fruit product purchasing behavior by introducing associative learning as a moderating variable. Experiments 1 and 2 examined how health-conscious and specific nutritional need purposes influenced preferences for fresh and processed fruits, while Experiment 3 revealed the important role of safety and taste preferences in consumer decision-making. Experiment 4 then explored the cognitive biases in consumers’ perceptions of processed fruits and how these biases influenced their preferences. By activating associative learning, negative perceptions of processed fruits were significantly heightened, especially for health-conscious consumers, who were more likely to choose fresh fruit. This experiment not only verified the moderating effect of associative learning but also reinforced the logical consistency and hierarchical structure of the previous experiments, demonstrating a multidimensional mechanism in consumer fruit product purchasing behavior under the influence of multiple factors.

### 4.2. Research Deepening and Practical Value

Based on research analyzing consumer preferences for fruits and their products, the study suggests that consumers’ eating purposes influence their willingness to purchase processed and unprocessed fruits. Guo Meilin et al. pointed out that perception behaviors often affect key psychological factors influencing consumers’ willingness to purchase organic foods. Health awareness drives them to pursue food free from pollution and chemical additives, while environmental concerns make consumers more inclined to products that promote ecological protection [52]. Additionally, the concept of food quality has expanded beyond traditional attributes such as taste and flavor, encompassing a broader multidimensional framework that includes food safety, nutritional or functional characteristics, and environmental impact [53,54,55,56]. This study constructs and verifies a series of hypotheses that reveal the complex mechanisms by which consumers’ consumption purposes (health-conscious and specific nutritional needs) influence preferences for fruits and their products. The study enriches consumer behavior research and offers a new perspective on understanding health awareness differences in food choices, providing a theoretical foundation for future food preference studies. Furthermore, it introduces safety and taste preferences as mediating variables and associative learning as a moderating variable, dissecting the roles of these psychological factors in consumer decision-making. The multidimensional analysis framework deepens the understanding of consumer food choice behaviors and provides a more comprehensive and detailed model for future research.

First, this research can help fruit producers and processors develop precise marketing strategies based on different consumer consumption purposes [57]. For consumers focused on dietary quality and health, it is important to highlight the natural nutrients in fruits, such as vitamins, minerals, and antioxidants, while promoting their natural, additive-free health benefits. For consumers with specific nutritional needs, such as the elderly or diabetic patients who prefer low-sugar or sugar-free dried fruits, or athletes and fitness enthusiasts who are more likely to choose high-energy, high-protein fruit products like fruit preserves, businesses can promote appropriate processed fruit products to meet these needs. By better understanding consumer health dietary needs, businesses can refine product positioning and marketing strategies to promote healthier eating behaviors.

Second, the study can help the fruit industry improve consumers’ awareness and acceptance of processed fruit products. By enhancing public education, improving product quality and safety, and addressing consumers’ concerns about the unhealthiness of processed fruits, businesses can increase consumer willingness to purchase these products. As the study concludes, consumer consumption purposes and preferences for fruit attributes align, but practical factors like seasonal fruit price changes or unmet taste preferences can make processed fruit products more desirable [58]. The fruit industry can seize this opportunity to attract more consumers by promoting the economic and nutritional value of processed fruit products, particularly during winter for tropical fruit products or summer for portable dried fruits as snacks for outdoor activities.

Lastly, this research emphasizes the importance of safety and taste preferences in consumer purchasing behavior. Providing fresh and safe fruits may not be enough to satisfy diverse consumer needs [59]. The fruit industry should consider consumers’ growing concerns about product quality and taste by demonstrating strict quality control in every step of production, from sourcing raw materials to processing and packaging. By using advanced food safety technologies to ensure fresh, uncontaminated fruits and monitoring harmful substances during processing, businesses can eliminate negative perceptions of processed fruits and better meet consumers’ health demands. This would foster sustainable development in the fruit industry, benefiting both economic and social outcomes [60].

## 5. Conclusions

This study systematically explores the impact of consumption purpose on consumer preferences for fruits and their products under the regulation of color perception. Through statistical analyses such as chi-square tests, Likert scales, and ANOVA, 489 urban Chinese consumers were surveyed. The results suggest that consumption purpose significantly influences preferences for fruits and their products. Health-conscious consumers tend to prefer fresh fruits with soft, coordinated colors, while consumers with specific nutritional needs are more inclined to purchase processed fruits with lower saturation or vivid hues. Additionally, the mediating variable of safety/taste preferences and the moderating variable of associative learning also affect consumer preferences. This research not only helps to gain a broader understanding of consumers’ attitudes towards green, healthy food but also contributes to guiding consumers in forming more scientifically reasonable eating habits, thus improving public nutritional health levels. It also provides scientific evidence for governments and related organizations to formulate food health policies and build a sustainable food consumption environment.

However, the study has some limitations. Although the sample population has diversity in terms of demographic characteristics, it is necessary to consider the potential impact of different natural environments and dietary habits on food consumption behavior, and the sample size should be further increased. Additionally, the research relied on online experiments with hypothetical purchasing scenarios, which may not fully capture real-world purchasing behavior and consumer willingness to buy. Future research could conduct integrated behavioral observations or choice experiments in real retail or simulated environments to further investigate consumers’ food consumption behavior in actual purchase situations, in order to enhance the ecological validity of the research.

## Figures and Tables

**Table 1:** Sociodemographic Description of the Sample Population.

Variable	Description	Frequency	Percentage (%)
Gender	Male	205	41.9
	Female	264	53.9
	Unknown	20	4.2
Age Group	22 years and below	55	11.2
	22–35 years	153	31.3
	36–50 years	179	36.6
	51 years and above	102	20.9
Education Level	High school or below	234	47.9
	Bachelor’s degree	162	33.1
	Graduate degree	93	19
Regular Fruit Consumption	Yes	462	94.5
	No	27	5.5
Location	Southern cities	325	66.5
	Northern cities	164	33.5
Health Status	Healthy	305	62.4
	Sub-healthy	184	37.6

**Table 2:** Common Fruits, Nutrients, and Their Functions.

Nutrient	Common Fruits	Effects
Vitamin A	Mango, Tomato, Watermelon, Banana, Peach, Persimmon	Reduces the likelihood of night blindness and vision impairment, promotes body development, strengthens bones, regulates skin and stratum corneum metabolism [36].
Vitamin B	Grapes, Banana, Kiwi, Tomato, Cherry	Protects the liver, maintains heart and vascular health, aids digestion and absorption of food, helps with fat loss.
Vitamin C	Kiwi, Lemon, Pineapple, Grapefruit, Orange	Whitening, antioxidant, promotes collagen synthesis, aids in the absorption of iron and folic acid, prevents iron-deficiency anemia, accelerates wound healing [37].
Vitamin E	Myrica, Apricot, Plum, Pomegranate, Blueberry	Protects cells from free radical damage, maintains skin health.
Carotene	Carrot, Peach, Orange, Apricot, Watermelon, Pineapple, Strawberry	Prevents, delays, and treats certain diseases, enhances the body’s immune function [38].
Lutein	Corn, Tomato, Banana, Orange, Plum	Antioxidant, helps protect the eyes and skin from UV damage.

**Table 3:** Experimental Selection and Features.

Attribute	Level	Experiment Key Features			
		Experiment 1	Experiment 2	Experiment 3	Experiment 4
Nutrient	Vitamin C	○●	○●		
	Vitamin B			○●	
	Vitamin E				○●
Fresh Fruit	Orange	○			
	Kiwi		○		
	Grapes			○	
	Blueberry				○
Processed Product	Canned Orange	●			
	Kiwi Preserve		●		
	Raisins			●	
	Blueberry Jam				●
Color Perception	Orange-yellow	○			
	Pale Yellow	●			
	Green		○	○	
	Yellow-green		●	●	
	Deep Blue				○
	Purple-black				●

“○” represents the unprocessed fresh fruit and characteristics of the experiment; “●” represents the processing of fruit products and experimental characteristics.

**Table 4:** Variable Definition.

Definition	Description	Variable Setting
Consumption Purpose	Health-oriented = 1; Specific nutritional needs = 2	Independent variable
Identification	Identified = 1; Unidentified = 2	Dependent variable

**Table 5:** Chi-Square Test Table for Consumption Purpose.

**Consumption Purpose**	**Identification**	**Frequency**
1	1	96
1	2	4
2	1	85
2	2	15

**Table 6:** Experiment 1 Consumption Purpose Manipulation Check Results Analysis.

Question	Name	Consumption Purpose (%)		Total	$X^{2}$	p
		Health-Oriented	Specific Nutritional Needs			
Identification	Identified	96 (48%)	85 (42.5%)	181 (90.5%)	7.037	0.014
	Unidentified	4 (2%)	15 (7.5%)	19 (9.5%)		
Total		100	100	200		

**Table 7:** One-Way ANOVA Results.

Consumption Purpose	Sample Size	Mean	Standard Deviation	95% Confidence Interval for Mean		*F*	p
				Lower Bound	Upper Bound		
Health-oriented	100	2.55	1.234	1.972	3.128	8.506	0.006
Specific Nutritional Needs	100	3.85	1.565	3.117	4.583		

**Table 8:** Analysis of the Manipulation Check Results for Eating Purpose in Experiment 2.

Question	Name	Consumption Purpose (%)		Total	$X^{2}$	p
		Health-Oriented	Specific Nutritional Needs			
Identification	Identified	105 (50%)	92 (43.8%)	197 (93.8%)	7.521	0.007
	Unidentified	12 (5.7%)	1 (0.5%)	13 (6.2%)		
Total		117 (55.7%)	93 (44.3%)	210		

**Table 9:** Analysis of the Manipulation Check Results for Fruit and Fruit Product Preferences in Experiment 2.

Consumption Purpose	Sample Size	Mean	Standard Deviation	95% Confidence Interval for Mean		*F*	p
				Lower Bound	Upper Bound		
kiwi	101	2.81	1.386	2.248	3.368	10.654	0.002
kiwi fruit preserves	89	4.21	1.475	3.499	4.921		

**Table 10:** Results of the Two-Way ANOVA in Experiment 2.

Source of Variation	Sum of Squares	df	Mean Square	F	p
Consumption Purpose	5.384	1	5.384	5.819	0.018
Fruit Attribute Preference	21.736	1	21.736	23.490	<0.001
Consumption Purpose × Fruit Attribute Preference	4.597	1	4.597	4.968	0.029

**Table 11:** Analysis of the Manipulation Check Results for Eating Purpose in Experiment 3.

Question	Name	Consumption Purpose (%)		Total	*X* ^2^	p
		Health-Oriented	Specific Nutritional Needs			
Identification	Identified	102 (51%)	80 (40%)	182 (91%)	5.025	0.025
	Unidentified	15 (7.5%)	3 (1.5%)	18 (9%)		
Total		117 (58.5%)	83 (41.5%)	200		

**Table 12:** Results of the One-Way ANOVA in Experiment 3.

Consumption Purpose	Sample Size	Mean	Standard Deviation	95% Confidence Interval for Mean		F	p
				Lower Bound	Upper Bound		
Health-oriented	117	2.93	1.507	2.371	3.496	13.229	<0.001
Specific Nutritional Needs	83	4.50	1.469	3.812	5.187		

**Table 13:** Mediation Model Test for the Relative Importance of Safety/Taste Preferences.

Category	Effect	*SE*	t	p	95%CI LL CI	UL CI
Direct Effect	0.119	1.632	0.073	0.942	−3.080	3.317
Indirect Effect	−3.040	1.121	−2.711	0.007	−5.238	−0.842

**Table 14:** Moderating Effect Model of Associative Learning Test.

Category	Effect	SE	*t*	*p*	95%*CI* LL CI	UL CI
Consumption Purpose (X)	3.7438	0.8838	4.2358	0.0001	1.9855	5.5020
Associative Learning (M)	4.8448	0.8434	5.7442	<0.05	3.1669	6.5226
X × M	−2.1101	0.5511	−3.8292	0.0003	−3.2063	−1.0139

## Data Availability

The original contributions presented in the study are included in the article, further inquiries can be directed to the corresponding author.
